# Derivation and Characterization of a ES-Like Cell Line from Indian Catfish *Heteropneustes fossilis* Blastulas

**DOI:** 10.1155/2014/427497

**Published:** 2014-01-20

**Authors:** Anindya S. Barman, Kuldeep K. Lal, Gaurav Rathore, Vindhya Mohindra, Rajeev K. Singh, Akankaha Singh, Praveen Khare, Bechan Lal

**Affiliations:** ^1^National Bureau of Fish Genetic Resources, Lucknow 226 002, India; ^2^College of Fisheries, CAU, Agartala, Tripura, India; ^3^Central Institute of Fisheries Education, Mumbai, India; ^4^Department of Zoology, Banaras Hindu University, Varanasi 221 005, India

## Abstract

A cell line designated as HFB-ES was established from blastula stage embryos of *H. fossilis* (Singhi). The embryonic cells were harvested and maintained in Leibovitz's medium supplemented with 15% fetal bovine serum. The cell line had been subcultured for more than 90 passages in a period of 24 months. HFB-ES cells were able to grow at temperatures between 25 and 35°C with an optimum temperature of 28°C. The growth rate of HFB-ES was proportional to FBS concentration, with optimum growth seen at 15% FBS concentration. The originality of the cell line was confirmed by sequencing of cytochrome oxidase c subunit I (COI), cytochrome b gene, and microsatellite DNA profile. Results of chromosome complements of HFB showed normal karyo-morphology with 56 (2*n*) diploid number of chromosomes after 40 passages which indicated that the developed cell line is chromosomally stable. The pluripotency of HFB was demonstrated by alkaline phosphatase activity and Oct-4 gene expression. Expression of GFP reporter gene was successful in HFB-ES. These results indicated that HFB-ES could be utilized for future gene expression studies.

## 1. Introduction

Development of mouse embryonic stem (ES) cells [1, 2] provided much of the technology for culturing ES cells derived from blastocysts. ES cells retain their development totipotency after even long-term culture and under specific conditions can differentiate into diverse cell types. The ability to manipulate these cells in culture allows generating site-directed mutations in their genome. They can be reintroduced into an early host embryo, which will colonize the embryo and contribute to *in vivo* formation of all three embryonic germ layers, including germ cells [1, 3].

Use of gene targeting techniques and development of ES cell lines have been done in small model fishes, such as zebrafish (*Danio rerio*) and medaka (*Oryzias latipes*). This is because they offer the possibility of combining embryological, genetic, and molecular analysis of vertebrate development. Recent [4, 5] review on the status and potential of embryonic stem cell research indicates that ES-like cell lines have been developed in medaka [6, 7], zebrafish [8, 9], the marine species *Sparus Aurata *[3], Sea bass [10], *Scophtalmus maximus *[11], *Paralichthys olivaceus* [12], and *Lateolabrax japonicus *[13]. In India, ES-like cell lines have been reported from Indian major carp, *Catla catla *[14].

The stem cells of embryonic origin can be useful for conserving diploid genome of threatened fish species [15]. The models of cloning through nuclear transfer [16, 17] enhance the utility of such cells equally for aquaculture improvement and conservation applications. These cells can also be used for genomic research for analyzing expression of important genes* in vitro *and* in vivo*. Considering the importance of ES cells, an effort was made to establish ES-like cells line from the blastula of Indian catfish,* H. fossilis *(HFB-ES), and characterize the qualities of pluripotent cells. This study happens to be the first successful attempt of culturing ES-like cells from a catfish species.

## 2. Materials and Methods

### 2.1. Collection of Embryonic Cells from Blastula Stage Embryo

**Figure 1:**
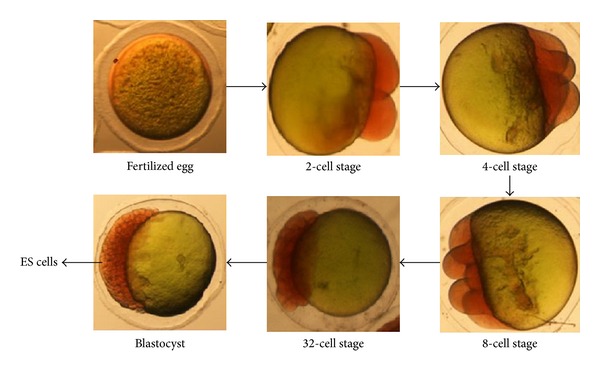
Developmental stages of *H. fossilis* embryonic cells (4x).

Blastula stage embryos of *H. fossilis* were harvested approximately 4 h after fertilization and prepared for cell culture. Approximately 150–200 embryos were disinfected with 70% ethanol and washed with phosphate-buffered saline (PBS) and the chorion was removed with pronase (0.5 mg/mL) for 15 min treatment. The embryos were then washed several times with L-15 medium to remove the cell membranes and single cells were obtained by gentle pipetting (Figure 1).

### 2.2. Primary Culture and Routine Maintenance

After several washes with PBS, the embryonic cells were transferred into 25 cm^2^ tissue culture flasks containing Leibovitz's (L-15) medium supplemented with 20% fetal bovine serum (FBS), 100 IU/mL penicillin, 100 *μ*g/mL streptomycin, and 2.5 *μ*g/mL fungizone. The flasks were incubated at 28°C and medium was replaced every 5 days. After the formation of a monolayer, the old medium was removed and cell sheets were washed twice with PBS and dispersed with 0.25% trypsin and 0.2% ethylene diamine tetra acetic acid (EDTA) in PBS. The cells were resuspended in 10 mL of growth medium and distributed into two flasks. The concentration of FBS in the L-15 medium was reduced to 15% for subcultures 20–40 and then to 10% in later subcultures. Culture was maintained for more than 90 passages in a period of 24 months and has been termed as HFB-ES cell line.

### 2.3. Evaluation of Growth Parameters of HFB-ES

The effects of temperature and FBS concentration on growth of HFB-ES were carried out at the 40th passage level. Cell culture flasks (25 cm^2^) were seeded with 10^5^ cells and incubated at 28°C for 2 h to allow for cell attachment. Batches of flasks were then incubated at selected temperatures of 25, 28, and 35°C for growth assessment. Every alternate day, duplicate flasks at each temperature were trypsinized for estimation of the cell density. The experiment was carried out for 10 days. Similarly, growth response of HFB-ES to different concentrations of FBS (5, 10, 15, and 20%) was also estimated at a constant temperature of 28°C.

### 2.4. Cryopreservation and Thawing of HFB-ES

HFB-ES containing 10^6^ cells in 1 mL was cryopreserved in liquid nitrogen at every 10th passage until 50th passage. Cryopreservation was done in L-15 medium containing 50% FBS and 10% DMSO as cryoprotectant. Viability and proliferation of HFB-ES were assessed after 7 days, 3 months, 5 months, and 12 months of cryopreservation. Viability of post-thaw cells was checked immediately as well as after 24 h of thawing.

### 2.5. Characterization of Cultured Embryonic Cells

#### 2.5.1. Molecular Confirmation of the Originality of HFB-ES

The origin of HFB-ES was confirmed by the use of microsatellites DNA marker and sequencing of partial regions of mitochondrial genes: cytochrome c oxidase subunit I (COI), cytochrome b, and 16S rRNA. For the confirmation, profiles for these molecular markers were generated from DNA obtained from HFB-ES and compared to DNA obtained from blood of *H. fossilis*.

#### 2.5.2. Use of Microsatellite Markers

The total genomic DNA from HFB-ES and ethanol-preserved blood of *H. fossilis* was extracted using reported protocol [18]. PCR amplification was performed (Thermal Cycler, Biorad Corp., USA, PTC 200) in a final volume of 25 *μ*L containing 1X PCR Buffer (10 mM Tris-HCl, pH9.0, 50 mM KCl, 0.01% gelatin), 1.5 mM MgCl_2_, 0.02 mM each dNTPs, 5 pmol primer, 1.5 units Taq DNA polymerase, and 50 ng of template DNA. Cycling conditions were initial denaturation at 94°C for 5 min followed by 35 cycles of denaturation at 94°C for 1 min, primer annealing at 50°C for 1 min, and primer extension at 72°C for 2 min with a final extension of 72°C for 2 min. Initially, 23 microsatellite primers were screened for the amplification with genomic DNA of *H. fossilis*. Out of these 23 primers, 3 microsatellite primers (Cb 3, Cb 13, and Cb 19) were selected to validate the origin of HFB-ES (Table 1). Amplified products were visualized on 10% native PAGE with silver staining. For interpretation of results, genotype profile of HFB-ES was compared with genomic DNA of *H. fossilis*.

### 2.6. Amplification and Sequencing of Mitochondrial Genes

Amplification of three partial mitochondrial DNA genes, cytochrome c oxidase subunit I (COI), cytochrome b and 16S rRNA genes, was performed using the genomic DNA obtained from both HFB-ES and fish blood. PCR conditions and primer details along with fragment specific annealing temperature are described in Table 2. PCR products were sequenced using Dye Terminator Cycle Sequencing Kit using fully automated capillary sequencer (MegaBace 1000, GE Healthcare Ltd., USA) as per manufacturer's protocol.

For comparing the sequence similarity of mitochondrial genes between HFB-ES and genomic DNA of *H. fossilis*, the sequences were aligned firstly using the ClustalW software [19] using default settings, resulting in an initial dataset, which was finally adjusted manually. The dataset was imported into software BIOEDIT [20] to construct dendogram using Neighbor-Joining (NJ) method based on gamma corrected Kimura-2 parameter distance matrices and displayed with TREEVIEW (version 1.66) [21]. Sequence homology of the genes with other fish species listed in NCBI database was also done with the help of nBLAST search.

### 2.7. Cytogenetic Confirmation of Originality of HFB-ES

Karyotyping of HFB-ES was carried out at passage 40 to determine the chromosomal stability and its origin. Briefly, the cells were seeded at a concentration of 1 × 10^6^/mL in 25 cm^2^ flask and incubated for 2 days until they reach logarithmic phase. Colchicines (Merck) were added to the cell culture medium for metaphase arrest at final concentration of 5 *μ*g/mL and flask was further incubated for 4–6 h. Cells were harvested and resuspended gently in 0.075 M KCl and incubated for 30 min at 25°C for hypotonic treatment. Cells were then fixed in freshly prepared and chilled coronary's fixative (3 methanol : 1 acetic acid), with three changes of 20 min each and then resuspended in a very small amount of fixative. The suspension was then dropped on cold moist glass slide and air-dried. Slides were then stained with 4% giemsa in phosphate buffer (pH 6.8) for 20 min and mounted with DPX medium. Metaphase spreads were examined under phase contrast microscope and images were captured at 100x resolution with the help of Leica CW4000 Karyo software (Leica Microsystems, Germany). The karyotypes of chromosome complements were prepared from cells exhibiting the complete somatic chromosome number and characteristic chromosome morphology. Homologous chromosomes were paired based on their morphology and position of centromere. The chromosome pairs were arranged based on morphology and decreasing order of size in the karyotype using Leica CW4000 Karyo software. Averages of the paired chromosomes were taken for estimating the length of short arm (p), long arm (q), arm ratio, centromeric index, and relative length (%). The arm ratio was used to classify the chromosomes as metacentric (m), submetacentric (sm), subtelocentric (st), and telocentric (t), as suggested by [22].

### 2.8. Exposure of HFB-ES to Retinoic Acid

To evaluate the differentiation potential of HFB-ES, 1 × 10^5^ cells were seeded into each of 24-well culture plates. Three days after seeding, medium was supplemented with 1 *μ*M of trans-retinoic acid (Sigma). The cells were grown for 5 weeks and compared with the control wells (without retinoic acid) to detect the differentiation potential of HFB-ES.

### 2.9. Oct-4 Gene Expression through Immunofluorescence Staining

Oct-4 gene expression was studied in HFB-ES by staining of the cells rabbit anti-Oct-4 polyclonal antibody (Chemicon, Millipore). The cells were grown for 24 h and were fixed in paraformaldehyde solution (4% for 20 min) at 4°C. Then washed with PBS, permeabilized with 0.1% Triton X-100 at 4°C for 4 min, and blocked in PBS containing 1% bovine serum albumin (BSA) for 30 min at room temperature. Thereafter, cells were incubated overnight at 4°C with the primary antibody at concentrations of 1 *μ*g/mL. The cells were washed and further incubated with secondary antibody; that is, FITC conjugated anti-rabbit IgG, at a 1/200 dilution for 1 hr. Cells were washed again and stained with DAPI and mounted with antifade 1,4-diazobicyclo-2,2,2-octanex (DABCO) in mounting medium (Sigma). Stained cells were visualized under fluorescence microscope (Nikon E-600; Nikon Corp., Japan).

### 2.10. Alkaline Phosphatase Staining of HFB-ES

The cells in logarithmic phase were harvested and fixed in glutaraldehyde solution (1%) for 10 minutes. The cell pellet was washed twice in PBS and stained for 20 min in dark for alkaline phosphatase activity using BCIP/NBT (Invitrogen) as substrate and was examined under the inverted microscope.

### 2.11. Expression of GFP Reporter Gene in HFB-ES

Transfection of GFP reporter gene was carried out in HFB-ES at passage 55 using preconstructed vector phMGFP (Invitrogen) by electroporation using electroporator (BioRad Ltd., USA). Preset protocol for electroporation of eukaryotic cells was followed for transfection. After the 24 h of transfection, cells were examined under fluorescence microscope (Nikon E-600, Nikon Corp., Japan).

## 3. Results

**Figure 2:**
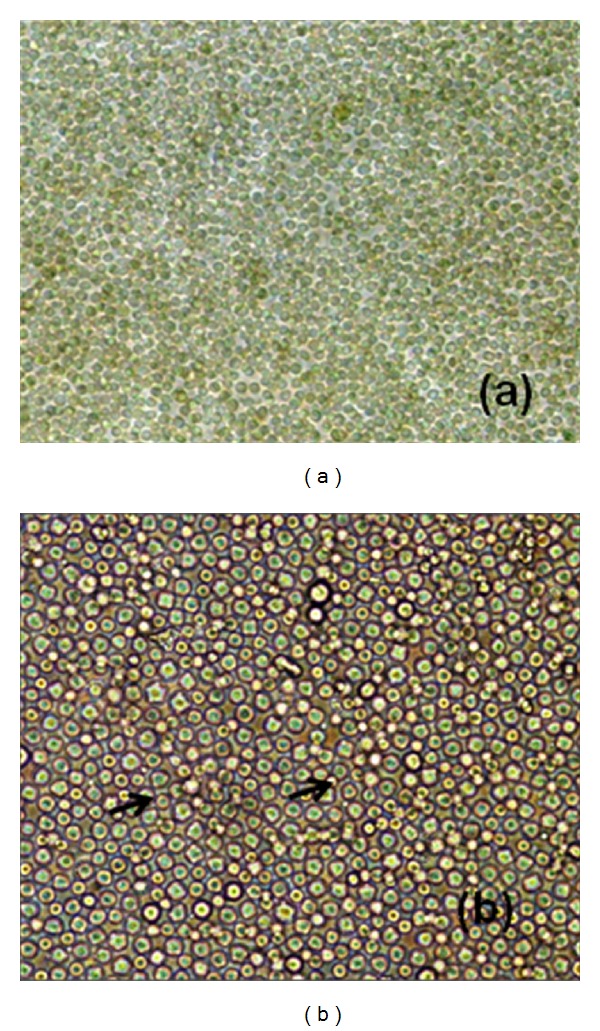
Development of embryonic cell line from blastula stage embryo of *H. fossilis*. (a) Confluent monolayer (10x). (b) Cells after 50 passages, showing polygonal cells (20x).

The *H. fossilis* oocytes were fertilized *in vitro*, which allowed following the development from cleavage up to blastua stage (Figure 1). Primary cell cultures were initiated from blastula stage embryo of *H. fossilis* by manual method. The cells grew well, formed a monolayer within 15 days, and were subcultured at intervals of 7–10 days. The cells were split in a ratio of 1 : 2. The primary culture consisted of round, oval shaped cells. Single cell suspension, confluent monolayer, and cell growth at different passages are shown in Figures 2(a) and 2(b).

**Figure 3:**
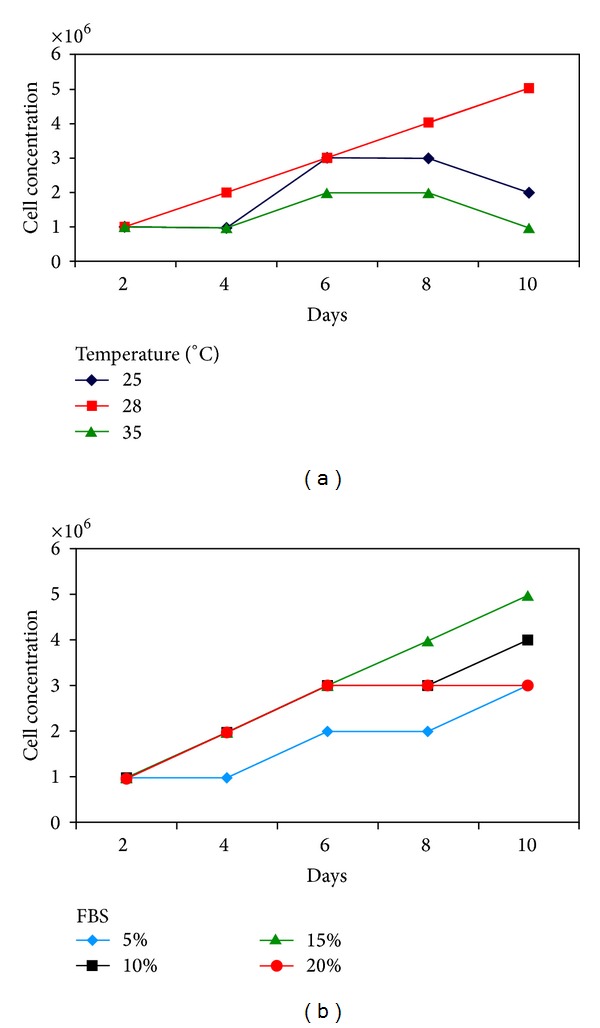
Evaluation of growth parameters of HFB-ES. (a) Effect of temperature. (b) Effect of FBS concentration (%).

HFB-ES exhibited different growths at different temperatures; these cells were able to grow at temperatures between 25 and 35°C and maximum growth obtained at 28°C (Figure 3(a)). The growth rate of the HFB-ES increased as the FBS proportion in the tissue culture medium was increased from 5 to 15% but decreased at 20% FBS. Maximum growth occurred with the concentrations of 15% FBS at 28°C (Figure 3(b)).

**Figure 4:**
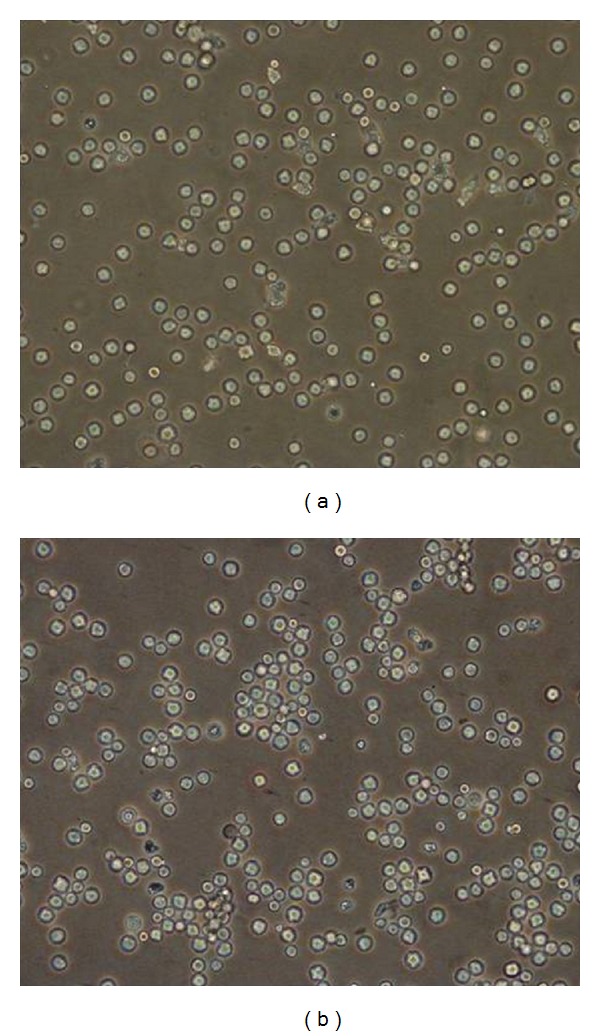
Embryonic cells cryopreserved (150 days). (a) Immediately after thawing. (b) Cryopreserved embryonic cells after 24 h of thawing. (10x).

Embryonic cells were cryopreserved with 50% FBS and 10% DMSO in liquid nitrogen at different passages. The post-thawed cryopreserved cells showed no visible morphological difference than the uncryopreserved cells and were able to proliferate in culture (Figures 4(a) and 4(b)).

**Figure 5:**
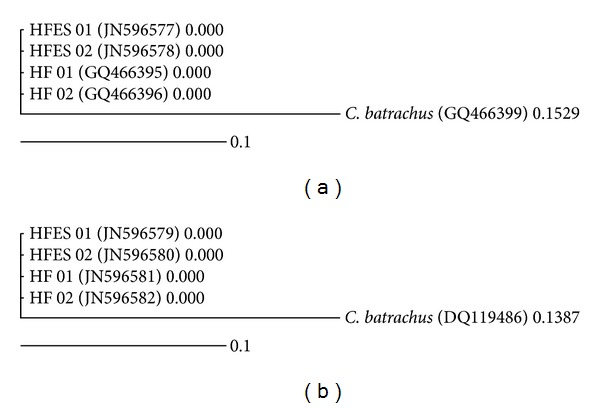
Confirmation of originality of cells: relatedness drawn between HFB-ES cells and *H. fossilis* based on the (a) partial COI sequences. (b) Cytochrome b mtDNA genes and *C. batrachus* sequences obtained from NCBI database were used as outgroup.

Microsatellite marker profiles and sequences of three mitochondrial genes, that is, COI and cytochrome b, validated the origin of HFB-ES cells. Three microsatellite marker primers were successfully amplified with genomic DNA isolated from HFB-ES and *H. fossilis* blood and showed similar microsatellite allelic patterns. Partial sequences of three genes, 655 bp of COI, 735 bp of cytochrome b and 540 bp of 16S rRNA, genes amplified from cultured cells, and *H. fossilis* blood were aligned with the help of ClustalW. The sequences from all the three regions exhibited 100% sequence homology between cultured cells and *H. fossilis* blood. The 16S rRNA gene sequences generated from embryonic cells also exhibited 99% similarity with the *H. fossilis* 16S rRNA sequences available in NCBI databank using nBLAST (sequence homology search tool). The dendograms based on the sequences of COI and cytochrome b genes obtained from cultured cells and *H. fossilis *blood genome were constructed along with the outgroup (*Clarius batrachus*) sequences downloaded from NCBI database for the same regions (Figures 5(a) and 5(b)). Tree topology computed from COI and cytochrome b genes showed single cluster for sequences from HF-like ES cells and fish blood DNA, while *C. batrachus* appeared as sister group with the distances of 0.1529 and 0.1387. Sequences of 16S rRNA gene were not used for dendogram construction due to high interspecies sequence similarity.

**Figure 6:**
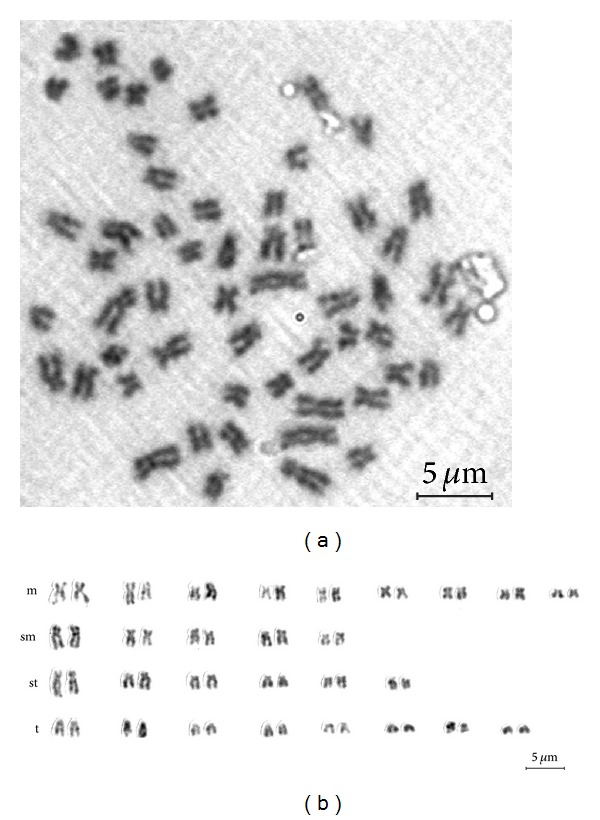
Karyotype of *H. fossilis* embryonic cell. (a) Metaphase spread and (b) karyotype, m = metacentric, sm = submetacentric, st = subtelocentric, and t = telocentric. Bar—5 *μ*m.

**Figure 7:**
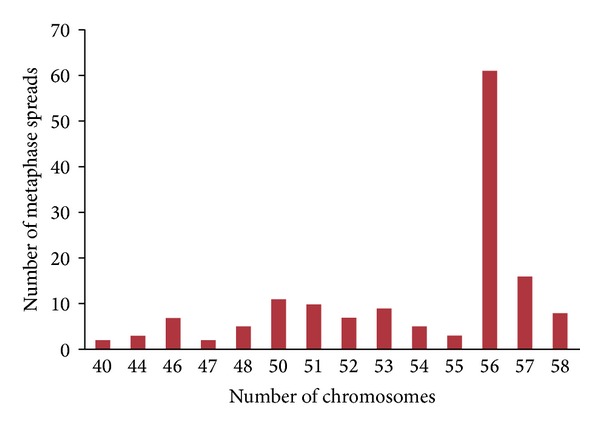
Frequency distribution of chromosomes in metaphase spreads of HFES cells.

The results of chromosome complement at passage 40 based on counts of 100 metaphase plates revealed that the number of chromosomes ranged from 40 to 58 (Figure 7) with modal number of chromosomes that was 28 pairs (2*n* = 56). Both heteroploidy and aneuploidy were observed in the cells, though they were small in proportion. The cells with 56 numbers of chromosomes displayed the normal karyotype morphology, consisting of 9 pairs of metacentric, 5 pairs of submetacentric, 6 pairs of subtelocentric, and 8 pairs of telocentric chromosomes (karyotype formula = 9 m + 5 sm + 6 st + 8 t with fundamental arm number FN = 42) (Figures 6(a) and 6(b)). Modal chromosome number and karyotype of the embryonic cell line are identical with the *H. fossilis* chromosome number. These data indicated that the HFB-ES cells have chromosomal stability.

**Figure 8:**
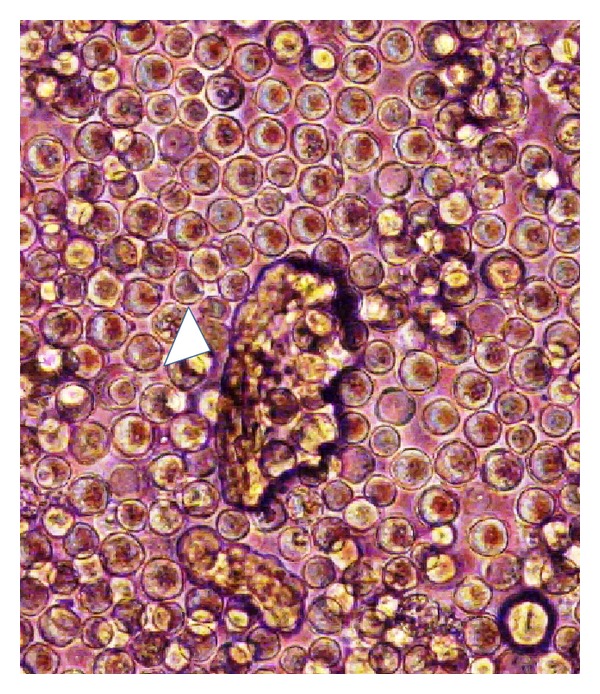
Embryoid body formation in embryonic cells of *H. fossilis* after retinoic acid treatment. White arrowheads showing embryoid bodies (100x).

Retinoic acid (vitamin A) is known to induce the differentiation of pluoripotent cells that result onto the formation of embryoid bodies. To identify the differentiation potential of HFB-ES, cells were treated with trans-retinoic acid and examined under microscope for the embryoid body formation after 3 weeks of incubation. Several embryoid bodies in treated cell culture (Figure 8) indicated the pluripotent differentiation property of the cultured embryonic cells.

**Figure 9:**
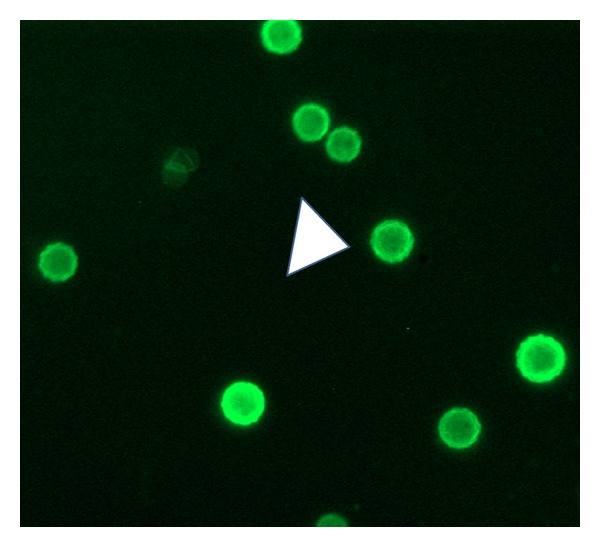
GFP expression in HFB-ES using phMGFP vector. Transfection was done with 2.0 *μ*g vector/100 *μ*L of cells. White arrowheads show the expression of GFP in transfected cells (100x).

**Figure 10:**
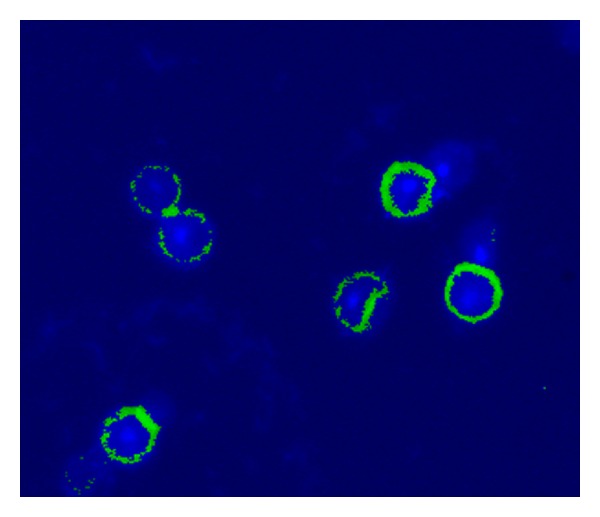
Immunofluorescence shows the expression of Oct-4 protein in embryonic cells. Blue colour dot showed the DAPI stained nucleus and green colour shows the expression of Oct-4 (1.5 *μ*g/mL concentration of Oct-4 antibody used) (100x).

**Figure 11:**
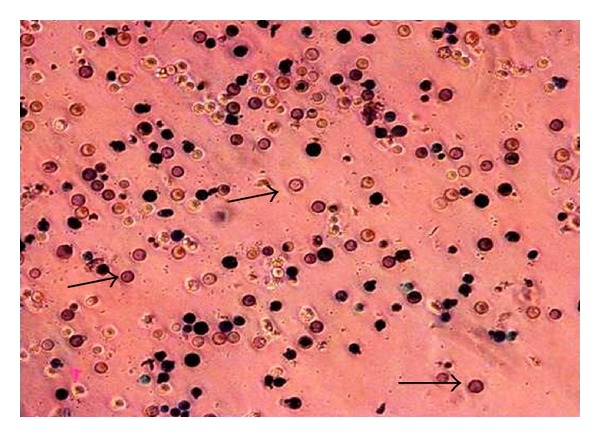
Alkaline phosphatase (AP) staining showed the pluripotency in embryonic cells (HFB-ES). White arrowheads show the purple coloured AP positive embryonic cells after 24 h of incubation (20x).

GFP expression was successfully observed in HFB-ES. The expression level of GFP was found to be the highest at the concentration of 2 *μ*g/100 *μ*L cells (Figure 9). Oct-4 expression was determined by immunofluorescence of the cells and best expression at 1.5 *μ*g/mL primary Oct-4 antibody concentrations (Figure 10). To study the pluripotency of the cells, alkaline phosphatase staining was carried out. Alkaline phosphatase is an enzyme that acts on aliphatic and aromatic phosphate esters and hydrolyzes them to release phosphates. After 24 h of incubation, more than 50% of cells showed alkaline phosphatase activity as they stained into bluish purple color in the presence of BCIP/NBT substrate (Figure 11). This finding depicted the pluripotency of embryonic cells.

## 4. Discussion

The present report describes the first ES-like cell line for any catfish species. The cell line had normal karyotype, exhibited renewal capacity and pluripotency as demonstrated through AP staining and was found useful for expression of transfected gene. Embryonic stem (ES) cells are pluripotent and thus able to differentiate into almost all cell types, providing an excellent system for experimental analyses of cellular and developmental events *in vitro *[26]. Their genetic manipulation allows targeted mutagenesis that can be transferred to the whole organism, by means of chimeras containing ES cells in the germ line [16].

The characterization of the HFB-ES cell line developed during the present study revealed that it confirmed to the majority of attributes described for ES cell line. These include renewal capacity for stable growth, a typical ES cell phenotype (a round or polygonal shape, a small size, large nuclei, and prominent nucleoli), high alkaline phosphatase activity, and a normal karyotype and the ability for spontaneous differentiation and can undergo clonal growth, forming compacted cell colonies of undifferentiated ES cells [5]. Similar characteristics had been reported in the ES-like cell lines developed for other fish species than zebrafish and medaka [7, 27, 28] as Seabream, Sparus, *Lateolabrax*, Japanese flounder, Asian sea bass, and other animals such as mice [1] and chicken [29].

The HFB-ES cells retained a normal karyotype, which was demonstrated after 40 passages of culture. Karyotype stability is the main property that should be preserved in ES cells for successful contribution into functional gametes. ES cell lines, like any other cell line kept in culture for long periods, are karyotypically unstable and can drift towards an aneuploid modal distribution of chromosomes [30]. The modal group of metaphases with 2*n* = 56, showed the standard chromosome morphology of this species (http://www.fishbase.org/). Chromosome values, below and above the diploid number, can be artifacts inherent to the technique. In mice, the number of culture passages had been found to correlate negatively with their ability to contribute to the chimeras and that aneuploidy increases with long-term ES cell cultures [30].

HFB-ES cell line maintained stable growth over the period of 24 months of culture with more than 90 passages and, however, interestingly and for unknown reasons HFB-ES cells remained as suspension rather than displaying adhesion. However, majority of the cell lines are known to grow with characteristics; yet growth in suspension has also been demonstrated [31]. This ES-like cell line could be easily grown and maintained in Leibovitz's L-15 medium, supplemented with fetal bovine serum at temperature (28°C). No specific growth factor was applied in the present work on the development of embryonic cell line of *H. fossilis*. In some of the studies growth factors, such as basic fibroblast growth factor (bFGF), have been applied to stimulate the proliferation of embryonic cells in zebrafish [32] and Japanese flounder [12]. The results clearly demonstrated that HFB-ES-like cell line grows over a wide range of temperature from 25 to 35°C with optimum growth at 28°C. The growth rate of HFB ES-like cells changed with FBS concentration increased from 5% to 20% and maximum growth was observed at 15%. Cryopreservation of cell lines is necessary for long-term storage. The feasibility of cryopreservation of this cell line was successfully demonstrated, and post-thaw recovered cells did not exhibit any visible morphological changes. These cells demonstrated their renewal capabilities, which is one of the characteristics of ES cell lines [4].

To confirm the cell line origin and to genetically identify the HFB-ES cell line, three microsatellite loci and sequencing of mitochondrial genes, COI (5′ region), partial cytochrome b, and 16S rRNA, were performed. Comparable pattern of amplified microsatellite loci and 100% sequence homology of mitochondrial genes between the HFB-ES cells and *H. fossilis* fish DNA confirmed the *H. fossilis* origin of the cell line. The cytogenetic screening of the HFB-ES cells also revealed an apparently euploid chromosome complement similar to what has been previously described in this species [33].

Alkaline phosphatase (AP) activity is high in totipotent cells *in vivo *and *in vitro*, but reduced or lost upon differentiation. The AP assay is routinely used to monitor the undifferentiated state of mouse stem cells in culture [10] and has been extensively used as a valuable marker of putative ES cells of many animal species including fish [6, 28]. In cultures with homogeneous cell population (typical ES-like morphology), all cells exhibited intensive staining for alkaline phosphatase, thus indicating its undifferentiated state and pluripotency. Additionally, this technique proved to be a useful marker for pluripotency in the *H. fossilis* all through the process of establishing ES cells, since high activity has been correlated with typical ES morphology and lower activity with differentiated cell morphologies [34].

The ability of the HFB-ES cells to produce embryoid bodies of undifferentiated cells *in vitro *also indicates pluripotent nature of these cells. The pluripotency of the HFB-ES cells was verified by the presence of Oct-4. Oct-4 is encoded by the POU domain transcription factor (POU5fl) and is essential for the development of inner cell mass (ICM) and required for the maintenance of pluripotent ES cells *in vitro *[35, 36]. The protein sequence, genomic organization, and chromosomal location of POU genes are highly conserved and probably not stringent species specific [37]. In an attempt to detect Oct-4 in *H. fossilis*, we evaluated an antibody raised against mouse Oct-4 and successfully confirmed the expression of Oct-4 in HFB-ES cells. However, similar to the observations in another teleost fish, Asian sea bass embryonic cell line [10], the activity was concentrated in cytoplasm instead of usually being found in nucleus in HFB-ES. The protein Oct-4 is present in the nuclei in all cleavage stages, but, following differentiation at the blastocyst stage, Oct-4 protein is only expressed in the inner cell mass [38]. Therfore, one of the possible explanations could be that the cells derived from blastocyst might have initiated differentiation and hence nuclear expression of Oct-4 was not evident in this study and the report on sea bass [10].

Due to lack of differentiation into various types of cells under RA treatment, the pluripotent nature of the HFB-ES is only partly demonstrated. Nevertheless, the *in vitro* characterization performed in the HFB-ES cell line, reflected so far the ES-like properties and could be grown for over 90 passages, thus looking very promising. In the present study, the cells were transfected with the phMGFP plasmid with GFP reporter. Successful transformation of the HFB-ES cells with the phMGFP plasmid and its apparently efficient expression represented important steps in the development process of ES cells in *H. fossils*. The ability of these cells to be transfected is very important, either to introduce a desired mutation to be transmitted in a mendelian way or, as an *in vivo* gene marker, to check the contribution of ES cells to the chimeras.

## 5. Conclusion

The pluripotent nature of the HFES cells is supported by their morphological features of round and polygonal cells, high alkaline phosphatase activity, Oct-4 expression, and the ability to remain undifferentiated for a prolonged culture period, the ability to form EBs, undergo differentiation spontaneously as a response to changes in the extracellular environment, and ability to transfect. These characteristics could make the HFB-ES cells a good experimental system for further *in vitro* studies of catfish embryo cell growth and differentiation.

## Figures and Tables

**Table 1:** Details of the primers used for the amplification of microsatellites.

S. number	Accession number	Locus	Sequence (5′-3′)	Number of alleles	Allele size range
1	AY169258	Cba03 F	ACAGCAGACATGCGTGTAAGGATT	1	129
		Cba03 R	TACTGTGGCTGTCAGAGCATC		

2	AY169268	*Cba*13 F	ACCAGGCGAGACGGAAGC	3	250–254
		*Cba*13 R	ACCTGCGCGGAGACGAGA		

3	AY169271	*Cba*19 F	CAGGGCTAAATTACCCATAATCA	7	215–255
		*Cba*19 R	GGCATGTGTTATAACATGTGAGG		

**Table 2:** Details of the primers used for the amplification of mtDNA genes.

mtDNA Gene	Primer ref.	Primer Sequence	Primer Ta
COI 655 bp	[23]	FishF1 5′-TGATCCGGCTTAGTCGGAACTGC-3′	50°C for 1 min
		FishR1 5′-GATGTGTGTTGAATTA CGGTCGG-3′	

Cyto b 735 bp	[24]	L14841 5′-AGCTTCCATCCAACATCTCAGCATG ATGA	50°C for 1 min
		H15149 5′-ACTGCAGCCCCTCAGAATGATATTT GTCCTC A-3′	

16S rRNA 485 bp	[25]	L2510 5′-CGCCTGTTTATCAAAAACAT-3′	53°C for 1 min
		H3080 5′-CCGGTCTGAACTCAGATCACGT-3′	
